# Integrated Analysis of Gut Microbiota and Metabolites in a Rat Necrotizing Enterocolitis Model

**DOI:** 10.5152/tjg.2025.24451

**Published:** 2025-01-20

**Authors:** Panjian Lai, Dayan Wang, Xiaobing Li

**Affiliations:** 1 Department of Pediatrics, Jinhua Maternal and Child Health Care Hospital, Zhejiang , China

**Keywords:** Gut microbiota, inflammatory cytokines, liquid chromatography-mass spectrometry, metabolites, necrotizing enterocolitis, 16S rDNA sequencing

## Abstract

**Background/Aims:**

Necrotizing enterocolitis (NEC) is a critical gastrointestinal emergency in newborns, characterized by mucosal damage, gut microbial dysbiosis, and inflammation. This study aimed to identify differential gut microbiota and metabolites in NEC and examine their correlations.

**Materials and Methods:**

A rat model of NEC was established using hypoxia–hyperoxia and cold stress. Histopathological analysis, liquid chromatography–mass spectrometry, and 16S rDNA sequencing were used to analyze intestinal contents and tissues. Proinflammatory cytokine concentrations were measured via enzyme-linked immunosorbent assay.

**Results:**

Necrotizing enterocolitis rats exhibited significant intestinal damage, including hemorrhage, edema, necrosis, and inflammatory infiltration (*P* < .05). Correlation analysis revealed strong associations between specific microbiota (e.g., *Lactobacillus* and *Morganella*) and metabolites, suggesting their involvement in NEC pathogenesis (*P* < .05). Furthermore, NEC rats had elevated proinflammatory cytokine levels, including interleukin-1β, tumor necrosis factor-α, and interleukin-6 (*P* < .05).

**Conclusion:**

This study successfully established a rat model of NEC, highlighting key microbiota–metabolite correlations and increased proinflammatory cytokines, providing insights into NEC pathogenesis and potential therapeutic targets.

**Main Points**

- The NEC rat model exhibited severe intestinal damage characterized by hemorrhage, edema, necrosis, and inflammatory infiltration.
- Significant associations were found between specific gut microbiota, particularly *Lactobacillus* and *Morganella*, and differential metabolites in NEC.
- Increased concentrations of proinflammatory cytokines, including interleukin-1β, tumor necrosis factor-alpha, and interleukin-6, were identified in NEC rats.
- The study provided novel insights into the pathogenesis of NEC and suggested potential targets for developing novel prevention and treatment strategies.

## Introduction

Necrotizing enterocolitis (NEC) is a life-threatening, common gastrointestinal emergency primarily impacting newborns, particularly those with very low birth weight.^1^ The pathological manifestations of NEC include uncontrolled acute inflammation, bacterial colonization, intestinal necrosis, and subsequent multi-organ damage.^2^ The prevalence of NEC is estimated to be around 7%,^3^ with a mortality rate of up to 20%-30%.^4^ Infants who survive NEC are at a high risk of developing long-term complications, including intestinal strictures and neurodevelopmental impairment.^5^ The development of NEC is associated with mucosal pathological changes in the immature intestine, which can be triggered by various factors, including microbial dysbiosis, bacterial overgrowth, and excessive inflammation.^6^ The use of biomarkers for the early identification of children with NEC has shown promising results.^7^

The human intestine contains trillions of microorganisms, mostly *Chlamydomonas* and *Bacteroidetes*, which play essential roles in multiple biological functions, such as host metabolism, immune regulation, maintaining the integrity of the intestinal mucosal barrier, vitamin synthesis, and neurodevelopment.^8^ Gut microbiota composition not only differs between individuals but also can be influenced by diet, environmental exposure, drugs, and diseases.^9^ In NEC, various factors, such as ischemia-reperfusion of intestinal tissues, release of inflammatory cytokines, microbial dysbiosis, and nutritional deficiencies in intestinal cells, can lead to intestinal mucosal barrier dysfunction, increased mucosal permeability, translocation of pathogenic bacteria in the intestine, and eventually intestinal failure.^10^ Analyzing gut microbiota and their metabolites may provide insights into the early diagnosis and treatment strategies for NEC.

Inflammatory cascade activation is considered the final common step in the pathogenesis of NEC.^11^ Reactive oxygen species (ROS)-mediated release of inflammatory cytokines (e.g., leukotrienes, tumor necrosis factor-alpha (TNF-α), and platelet-activating factor) has been found to induce intestinal mucosal damage in NEC.^12^ Elevated levels of inflammatory cytokines are associated with an elevated risk of late neurological damage in pediatric NEC patients.^13^ Furthermore, it has been reported that gut microbiota and their metabolites can modulate inflammatory pathways and immune responses in NEC by interacting with intestinal epithelial cells and immune cells.^14^ Recent studies^12,13^ have demonstrated that the gut microbiota plays a crucial role in the pathogenesis of NEC, with certain genera being implicated more frequently in disease progression. *Lactobacillus*, while typically associated with gut health, has been reported to behave differently in the context of intestinal dysbiosis, with some strains contributing to inflammation and barrier dysfunction depending on the gut environment. Its involvement in NEC has been found in both protective and pathogenic roles, making it a relevant target for further investigation.^9^ In contrast, *Morganella*, a genus known for its pathogenicity, is commonly linked to sepsis and inflammatory conditions in the gastrointestinal tract, particularly under compromised intestinal conditions, such as NEC. Previous studies^11 12^ have highlighted its association with increased epithelial damage and inflammatory responses. These findings, together with our correlation analysis, justify the concentration on these genera in our study as key contributors to NEC pathogenesis.

In this study, a rat model of NEC was developed to identify key differences in gut bacteria and metabolites between the NEC and control groups. The correlations between gut bacteria and metabolites were also explored, and the levels of inflammatory cytokines were compared between the intestines of the NEC and control groups.

## Materials and Methods

### Necrotizing Enterocolitis Rat Model and Grouping

Forty Sprague–Dawley neonatal rats (Hangzhou Hebei Technology Co., Ltd., Hangzhou, China) were randomly allocated into 2 groups: the Control group, which received routine feeding without intervention, and the NEC group, which was fed enteral formula and subjected to a combination of hypoxia–hyperoxia and cold stress. This involved exposing the newborn rats to 100% CO_2_ for 10 minutes, 4°C for 5 minutes, and 97% O_2_ for 5 minutes twice a day for 3 days, along with saline injection into the peritoneal cavity. Subsequently, all rats were euthanized by intraperitoneal injection of pentobarbital sodium at a dose of 150 mg/kg to induce respiratory arrest. Intestinal contents were obtained from each animal by gently squeezing the end of the rectum near the base of the tail, collected in sterile EP tubes, and kept at –80°C for further analyses. The entire intestine (from the distal end of the duodenum to the cecum) was removed, washed with sterile water, and kept at –80°C. The NEC model used in this study was based on established protocols that replicate NEC-like intestinal injury in neonatal rats, as described in previous research.^15^ This model involves the combined use of hypoxia–hyperoxia and cold stress, which has been demonstrated to induce NEC-like symptoms, including intestinal inflammation, necrosis, and dysbiosis, mimicking the pathophysiology of NEC observed in human infants.^15^ This study was approved by the Experimental Animal Ethics Committee of Jinhua Food and Drug Inspection and Testing Research Institute China (approval no. AL-JSYJ202024, Date: May 31, 2020). All methods were performed in accordance with relevant guidelines and regulations, and informed consent was obtained from all relevant parties.

### Histopathological Examination

Intestinal tissues, specifically from the ileum, were fixed in 4% formaldehyde solution for 3-5 days, followed by dehydration in ethanol. Subsequently, the tissues were paraffin-embedded and cut into 4-μm thick slices. Hematoxylin and eosin (H&E) staining was then performed by immersing tissue sections in the hematoxylin staining solution (cat. no. H9627, Sigma-Aldrich, USA) and eosin (cat. no. E6003, Sigma-Aldrich) staining solution, respectively, for 5 minutes. After dehydration, sections were observed under an Olympus light microscope (Model: BX43; Japan).

### 16S rDNA Sequencing

DNA Extraction Kit (vendor information) was used to extract genomic DNA from the intestinal contents. Polymerase chain reaction (PCR) primers were designed based on the 16S rDNA gene sequence of each bacterium using Primer-Express v3.0 software. The designed primer sequences were validated by comparing them with the full sequences in the BLAST database (www.ncbi.nlm.nih.gov/BLAST). The genomic DNA extracted from the intestinal contents was amplified by conventional PCR. The reaction system was composed of 10 μL of 2× Taq Plus Master Mix (vendor information), 1 μL of forward primer, 5 μL of DNA template, 1 μL of reverse primer, and 3 μL of deionized distilled H_2_O. The PCR reaction conditions consisted of an initial pre-denaturation at 95°C for 5 minutes and 30 cycles of denaturation at 95°C for 40 seconds, annealing at 52°C for 30 seconds, and extension at 72°C for 40 seconds. After the last denaturation step at 72°C for 10 minutes, the products were separated through agarose gel (2%) electrophoresis, purified, and subsequently sequenced. The obtained sequencing data were used to identify the microbial species present in the intestinal contents.

### Liquid Chromatography–Mass Spectrometry

Intestinal metabolites were detected using liquid chromatography–mass spectrometry (LC–MS). The intestinal content samples (50 mg) were combined with cold methanol (400 μL; methanol : water = 4 : 1) and mixed thoroughly at low temperature. Next, the samples were subjected to 3 cycles of ice-cold ultrasonic extraction, each lasting 10 minutes. After sitting at –20°C for half an hour, the samples were centrifuged at 4°C, 13 000 g for 15 minutes. The supernatants were then collected for LC–MS (UPLC-TripleTOF, AB SCIEX) using the 1.7-μm BEH C18 column (100 mm × 2.1 mm; Waters, Milford, USA). Eluent A was composed of water containing 0.1% formic acid, while eluent B was a mixture of isopropanol and acetonitrile (1/1) supplemented with 0.1% formic acid. The gradient elution was set as follows: 0-3.0 minutes, 0%-20% B; 3.0-9.0 minutes, 20%-60% B; 9.0-11.0 minutes, 60%-100% B; 11.0-13.5 minutes, 100% B; 13.5-13.6 minutes, 100%-0% B; 13.6-16.1 minutes, 0% B. During the analysis, the flow rate was set at 0.40 mL/min, with the injection volume of 20 μL and the column temperature of 40°C. The sample mass spectra were obtained in both negative and positive ion scanning modes. The collision, electrospray capillary, and injection voltages were set at 6 eV, 1.0 kV, and 40 V, respectively. The ion source temperature was held at 120°C, while the desolvation temperature was fixed at 500°C. The carrier gas flow rate was 900 L/h. Mass spectra were scanned with a resolution of 3000 in the range of 50-1000 m/z.

### Enzyme-Linked Immunosorbent Assay

Intestinal tissue homogenates were generated and centrifuged to isolate the supernatant. The concentrations of inflammatory cytokines (i.e., interleukin 6 (IL-6), TNF-α, and interleukin-1β (IL-1β)) in the rat intestine were determined using enzyme-linked immunosorbent assay (ELISA) kits (Liabio, Shanghai, China). The optical density value was measured using the Spectra Max Plus 384 microplate reader (Molecular Devices, USA) at a wavelength of 450 nm.

### Statistical Analysis

Statistical analyses were conducted using SPSS 22.0 (IBM SPSS Corp.; Armonk, NY, USA). Measurement data were expressed as mean ± SD. In cases where data exhibited a normal distribution, an independent *t*-test was employed to compare the means between groups. When data did not follow a normal distribution, a rank sum test was utilized. Correlation heatmaps, correlation chord plots, correlation network diagrams, and correlation Sankey diagrams were generated to explore the association between significantly different microbes at the genus level and significantly different metabolites between the NEC and control rats. Statistical significance was defined as *P *< .05.

## Results

### Intestinal Damage in Rats with Necrotizing Enterocolitis

**Figure 1.**
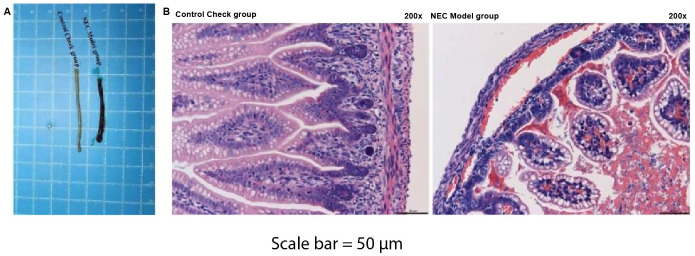
Histopathological changes in the intestinal tissues of necrotizing enterocolitis rats. Sprague–Dawley neonatal rats were randomly divided into control and necrotizing enterocolitis groups (n = 20 per group). (A) Representative macroscopic images of the intestinal tract from both groups. (B) Histopathological evaluation of the intestinal tissues using hematoxylin and eosin staining (200× magnification).

An in vivoNEC model was constructed by exposing rat neonates to a combination of hypoxia–hyperoxia and cold stress. The entire intestine and intestinal contents were then collected from both the NEC group and control animals. The macroscopic images of the gut revealed significant differences in gut color and dilatation between the 2 groups (Figure 1A). The healthy intestine displayed a smooth surface and consistent color throughout, with no signs of abnormal dilatation. In contrast, NEC rats exhibited distinct alterations in the macroscopic appearance of the gut. The gut color was altered, accompanied by signs of dilatation and discoloration, which collectively indicated significant pathological changes (*P* < .05). Further histopathological analysis using H&E staining confirmed significant changes in the intestines of model rats, including hemorrhage, edema, extensive necrosis, blurred structure, and inflammatory infiltration (*P* < .05). The control group, however, displayed clear and intact intestinal tissues without any lesions, indicating a healthy state (Figure 1B). These findings indicate severe damage and disruption of the intestinal tissues in the NEC model group.

### Correlations between Gut Microbiota and Metabolites

**Figure 2.**
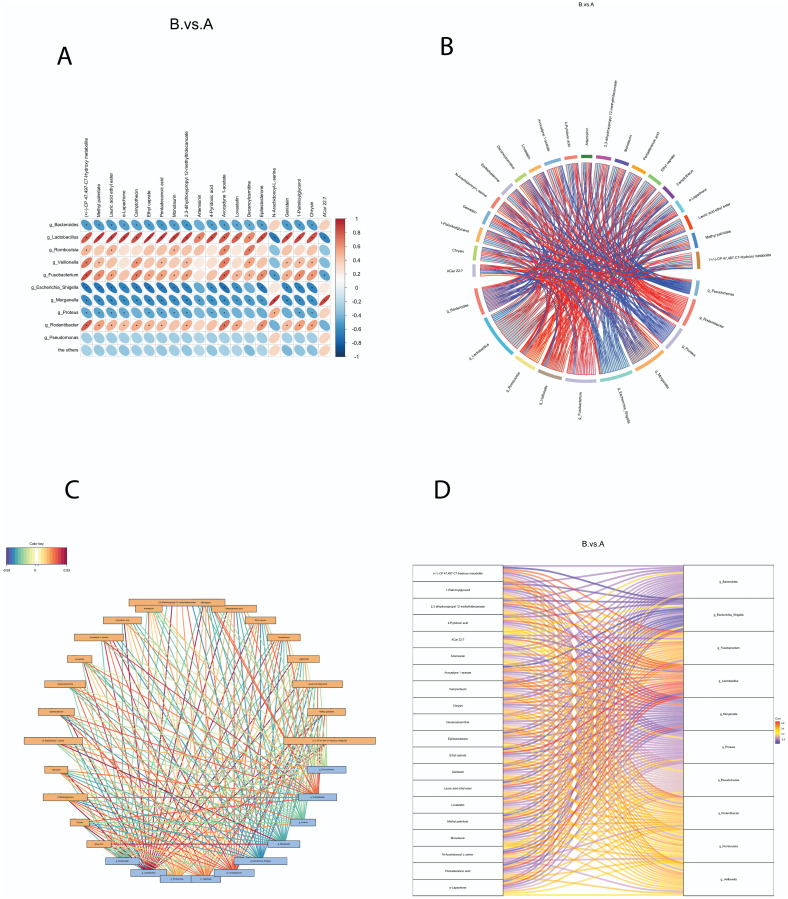
Correlation between gut microbiota and metabolites in positive ion mode. Gut microbiota in rat intestinal contents were identified via 16S rDNA sequencing, and intestinal metabolites were analyzed using liquid chromatography* –*mass spectrometry in positive ion mode. (A) Heatmap of Pearson’s correlation coefficients (ranging from −1 to 1), with positive correlations in red and negative correlations in blue. Significant correlations (*P* < .05) are marked with asterisks (*). (B) Chord plot showing the strength of correlations between microbiota and metabolites; wider chords indicate stronger correlations (red for positive, blue for negative). (C) Correlation network diagram where blue boxes represent microbiota, yellow boxes represent metabolites, and connecting lines indicate the direction and strength of the correlations (red = positive, blue = negative). (D) Sankey diagram depicting microbiota–metabolite correlations, with differential metabolites on the left and differential microbiota on the right (red = positive, blue = negative).

**Figure 3.**
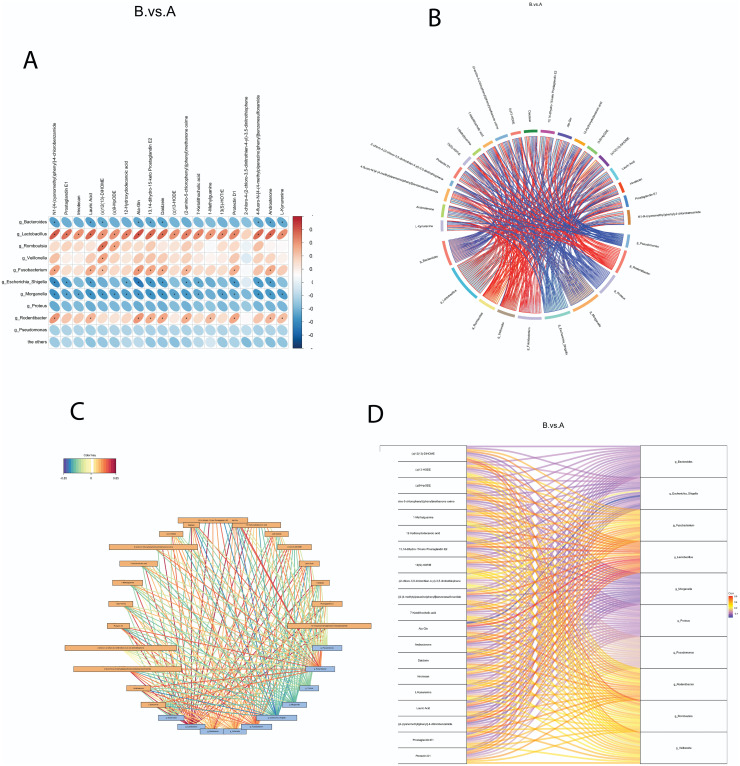
Correlation between gut microbiota and metabolites in negative ion mode. Microbial species in rat intestinal contents were identified by 16S rDNA sequencing, and metabolites were analyzed using liquid chromatography* –*mass spectrometry in negative ion mode. (A) Heatmap of Pearson’s correlation coefficients (from −1 to 1), with red indicating positive correlations and blue indicating negative correlations. Significant correlations (*P* < .05) are denoted by asterisks (*). (B) Chord plot illustrating the strength of correlations between microbiota and metabolites; wider chords indicate stronger correlations (red = positive, blue = negative). (C) Correlation network diagram with microbiota (blue boxes) and metabolites (yellow boxes), with connecting lines showing correlation direction and strength (red = positive, blue = negative). (D) Sankey diagram visualizing microbiota–metabolite correlations, with differential metabolites on the left and microbiota on the right (red = positive, blue = negative).

To explore the relationships between different microbiota and metabolites in the intestinal contents, we performed correlation analysis using the top 20 different metabolites and the top 10 different microbiota identified between the control and NEC groups. The correlation heatmaps presented in Figures 2A and 3A visually demonstrated the associations determined by Pearson correlation coefficient analysis. The significance of the correlations was confirmed with *P*-values < .05, providing a comprehensive overview of the relationship between metabolites and microbiota. To further explore their correlations, we generated correlation cohort plots and network diagrams, which revealed multiple and interconnected connections between the metabolites and microbiota. The positive ion method was used for Figure 2B and C, while the negative ion method was employed for Figure 3B and C. To provide a more intuitive representation of the associations between gut microbiota and metabolites, Sankey charts were plotted. These charts, depicted in Figure 2D for the positive ion method and Figure 3D for the negative ion method, visually display the complex microbiota–metabolite network. Upon analyzing the correlation results, we found that *Lactobacillus* and *Morganella *exhibited significant correlations with all metabolites in the positive ion mode (*P* < .05). *Bacteroides*, *Fusobacterium*, and *Escherichia–Shigella* were significantly correlated with most metabolites, such as (±)-CP 47,497-C7-hydroxy metabolite and avocadyne 1-acetate (*P* < .05). In the negative ion mode, *Lactobacillus*, *Morganella*, and *Escherichia–Shigella *showed significant correlations with most metabolites (*P* < .05). These findings collectively unveil the key microbiota–metabolite pairs in the control and NEC animals, indicating their potential roles in modulating the pathogenesis of NEC.

### Upregulation of Inflammatory Cytokines in the Intestine of Necrotizing Enterocolitis Rats

**Figure 4.**
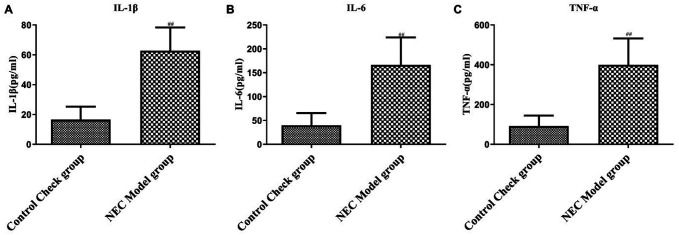
Inflammatory cytokine levels in rat intestinal tissues. Concentrations of (A) tumor necrosis factor-alpha, (B) interleukin-6, and (C) interleukin-1β were measured using enzyme-linked immunosorbent assay. *P* < .01.

Lastly, we assessed the concentrations of proinflammatory cytokines in the intestinal tissue samples of both groups. The NEC model group exhibited a notable increase in TNF-α (Figure 4A, *P* < .05), IL-6 (Figure 4B, *P* < .05), and IL-1β (Figure 4C, *P* < .05) levels in the intestinal tissues compared with the control animals. The elevated concentrations of these cytokines indicate the presence of NEC-induced inflammatory responses in the intestine, emphasizing the involvement of inflammatory factors in the pathogenesis of NEC.

## Discussion

In the initial stage of the disease, it can be challenging to differentiate NEC from other conditions frequently observed in premature infants solely based on clinical manifestations, and this highlights the necessity for distinct disease biomarkers.^15^ Although the exact pathogenic mechanisms are not completely understood, there is a prevailing belief that the developing gut microbiota and abnormal immune responses to feeding are essential to NEC development.^16^ In this work, we explored the associations between the top different gut microbiota and metabolites in neonatal rats with NEC compared to healthy control animals. Our findings revealed significant correlations of *Lactobacillus* and *Morganella *with a majority of the top different metabolites, providing novel perspectives on the pathogenesis of NEC.

*Lactobacillus*, one of the most important probiotic bacteria that belongs to the phylum *Firmicutes*, plays a vital role in the gut microbiome by maintaining gut barrier integrity and aiding in the defense of the mucosal barrier and modulation of host immune response.^17^ The gut-residing *Lactobacillus* not only serves as a protective barrier against pathogen attachment and survival within the gastrointestinal tract but also mediates both adaptive and innate immune responses by promoting the differentiation of immune cells, such as T cells and macrophages.^18^ Additionally, *Lactobacillus* and their metabolites have been shown to mitigate metabolic disorders by suppressing inflammatory responses and alleviating oxidative stress.^19^ An *ex-vivo *investigation involving human intestinal cells extracted from the ileum of patients with NEC showed that treatment with *Lactobacillus rhamnosus* markedly diminished the activation of inflammatory pathways.^20^ Furthermore, a meta-analysis of 23 placebo-controlled, randomized clinical trials reported that *Lactobacillus* supplementation effectively lowered the occurrence of NEC and mortality rate in premature infants, without increasing the occurrence of sepsis.^21^ Our data revealed that *Lactobacillus *exhibited positive correlations with most metabolites in the negative ion mode and all metabolites in the positive ion mode, which confirmed *Lactobacillus *as a key regulator during the pathogenesis of NEC.

*Morganella*, a Gram-negative bacteria genus within the family *Enterobacteriaceae*, is commonly found in the intestinal tracts of mammals and humans.^22^ It is widely recognized as an opportunistic pathogen that can induce a diverse range of clinical and community-acquired infections, including chorioamnionitis, cellulitis, and sepsis, resulting in significant mortality and morbidity.^23^ A rapid elevation in the abundance of *Morganella morganii *has been observed in association with various pathological conditions, such as trauma, ischemia, hypoxia, and inflammation.^24^ The presence of *M. morganii *has also been reported in leukemic children with NEC.^25^ Moreover, during the initial 4 weeks of life, NEC infants showed notably higher abundance of *M. morganii* compared to control infants.^26^ In this study, we further demonstrated that *Morganella *was negatively correlated with all metabolites in the positive ion mode and with the majority of metabolites in the negative ion mode, confirming the close implication of *Morganella *in the development of NEC.

The imbalance between protective anti-inflammatory cytokines and proinflammatory mediators is considered a key contributor to intestinal dysbiosis in NEC.^27^ An increased expression of IL-6, TNF-α, and IL-1β has been reported in murine NEC intestines.^28^ Moreover, Li and Sheng^29^reported a significant elevation in the serum concentrations of TNF-α and IL-6 in neonates with NEC, with the peak observed at 24 hours. Similarly, we showed that these proinflammatory factors were significantly upregulated in the intestines of NEC rats versus the control animals. Guo et al^30^ found that administration of *Lactobacillus acidophilus* alleviated NEC symptoms, reduced intestinal epithelial apoptosis, and decreased the TNF-α and IL-6 levels in a rat NEC model. Further exploration is warranted to investigate how other gut bacteria and their metabolites modulate inflammatory pathways in NEC.

The gut microbiota plays a pivotal role in modulating inflammatory responses by regulating cytokine production. Previous studies^23 24^ have demonstrated that specific microbial species can either exacerbate or attenuate the inflammatory processes associated with NEC. For instance, *Lactobacillus* species have been reported to decrease the expression levels of proinflammatory cytokines, such as TNF-α, IL-6, and IL-1β, through mechanisms involving the regulation of Toll-like receptors (TLRs) and the modulation of immune cell differentiation. In a study by Guo et al,^30^ *L. acidophilus* administration significantly reduced the levels of TNF-α and IL-6 in a NEC rat model, leading to decreased intestinal epithelial apoptosis and alleviation of NEC symptoms. This suggests that *Lactobacillus* may act as a protective agent by mitigating inflammatory responses in the gut. Conversely, pathogenic bacteria, such as *Morganella*, have been associated with elevated levels of TNF-α and IL-1β in the context of intestinal inflammation. Increased abundance of *M. morganii* has been linked to elevated inflammatory responses in NEC, driving the excessive cytokine production that contributes to the progression of tissue damage.^25^ In the present study, significant correlations were found between *Morganella* and metabolites associated with inflammatory pathways, further highlighting the role of pathogenic bacteria in promoting the inflammatory cascade observed in NEC. The differential modulation of cytokines by various microbial species suggests that the gut microbiota composition could be a key determinant in the balance between proinflammatory and anti-inflammatory responses in NEC.

In conclusion, a rat NEC model was successfully established, demonstrating significant morphological and pathological alterations in intestinal tissues. The macroscopic examination revealed notable differences between the NEC and control groups, with the NEC group exhibiting marked changes in gut color, dilatation, and signs of tissue damage (*P* < .05). Histopathological analysis further confirmed extensive damage characterized by hemorrhage, edema, necrosis, and inflammatory infiltration in the NEC model, contrasting with the intact and healthy intestinal architecture observed in the control group (*P* < .05). Additionally, the correlation between gut microbiota and metabolites was significant, particularly with the top differential gut microbiota, including *Lactobacillus* and *Morganella*, which were found to be correlated with most of the identified metabolites in both positive and negative ion modes (*P* < .05). Notably, *Bacteroides, Fusobacterium*, and *Escherichia–Shigella* were also significantly associated with key metabolites, indicating their potential roles in the biochemical pathways related to NEC pathogenesis. Furthermore, the assessment of pro-inflammatory cytokines indicated that the NEC model group had elevated levels of TNF-α, IL-6, and IL-1β (*P* < .05), underscoring the inflammatory response associated with NEC. Overall, this study not only validated the rat model for NEC but also elucidated the relationships among gut microbiota, metabolites, and inflammatory cytokines, suggesting that *Lactobacillus* and *Morganella* could serve as potential biomarkers for NEC. These insights could facilitate the development of novel prevention and treatment strategies, aiming to modulate gut microbiota and inflammatory responses to mitigate the risks and impacts of NEC in vulnerable populations.

## Data Availability

The datasets generated and/or analyzed during the current study are available in the Figshare repository: Raw data of 16S rDNA sequencing: https://figshare.com/articles/media/_b_Integrated_analysis_of_gut_microbiota_and_metabolites_in_a_rat_necrotizing_enterocolitis_model_b_/25705938; Raw data of metabolites: https://figshare.com/articles/thesis/_b_Integrated_analysis_of_gut_microbiota_and_metabolites_in_a_rat_necrotizing_enterocolitis_model_b_2_/25674021
